# *In Vitro* Effect of* Cinnamomum zeylanicum* Blume Essential Oil on* Candida* spp. Involved in Oral Infections

**DOI:** 10.1155/2018/4045013

**Published:** 2018-10-17

**Authors:** Marianne de Lucena Rangel, Sabrina Garcia de Aquino, Jefferson Muniz de Lima, Lúcio Roberto Castellano, Ricardo Dias de Castro

**Affiliations:** ^1^Graduate Program in Dentistry, Federal University of Paraíba (UFPB), 58051-900 João Pessoa, PB, Brazil; ^2^Human Immunology Research and Education Group (GEPIH), Technical School of Health (Escola Técnica de Saúde, Universidade Federal da Paraíba), UFPB, 58051-900 João Pessoa, PB, Brazil

## Abstract

The present study demonstrates the antifungal potential of chemically characterized essential oil (EO) of* Cinnamomum zeylanicum *Blume on* Candida* spp. biofilm and establishes its mode of action, effect on fungal growth kinetics, and cytotoxicity to human cells. The minimal inhibitory concentration (MIC) and minimal fungicidal concentration (MFC) values varied from 62.5 to 1,000 *μ*g/mL, and the effect seems to be due to interference with cell wall biosynthesis. The kinetics assay showed that EO at MICx2 (500 *μ*g/mL) induced a significant (p < 0.05) reduction of the fungal growth after exposure for 8 h. At this concentration, the EO was also able to hinder biofilm formation and reduce* Candida* spp. monospecies and multispecies in mature biofilm at 24 h and 48 h (p < 0.05). A protective effect on human red blood cells was detected with the EO at concentrations up to 750 *μ*g/mL, as well as an absence of a significant reduction (p > 0.05) in the viability of human red blood cells at concentrations up to 1,000 *μ*g/mL. Phytochemical analysis identified eugenol as the main component (68.96%) of the EO.* C. zeylanicum *Blume EO shows antifungal activity, action on the yeast cell wall, and a deleterious effect on* Candida* spp. biofilms. This natural product did not show evidence of cytotoxicity toward human cells.

## 1. Introduction

*Candida* spp. are commensal versatile microorganisms that colonize up to 70% of dental prosthesis users. However, local or systemic alterations might cause an imbalance in the host, predisposing the host to the development of disease, which might range from superficial and localized involvement to a fatal condition when it disseminates across the body of immunocompromised individuals [1]. The oral manifestations of the disease are considered signs indicative of an immune response imbalance, particularly among patients with acquired immunodeficiency syndrome [2].

Essentially four classes of drugs exist for the treatment of fungal infections with* Candida*: polyenic agents, azoles, echinocandins, and antimetabolites. As oral infections are superficial, treatment includes topical antifungals, such as nystatin and miconazole. Despite the number of available therapeutic options, microbial resistance to antifungals from different classes is increasing [3–7]. One of the mechanisms strongly associated with such resistance is the ability of certain microorganisms to form biofilms. Microorganisms in these communities undergo genetic changes that increase their resistance to drugs and the immune system. In the case of* Candida*, its ability to form biofilm on biotic and abiotic surfaces represents a relevant virulence factor and is thus an interesting target of study of the development of antifungal agents against candidiasis [8–11].

Within this context, the study of the therapeutic properties of plants that may represent an alternative therapeutic resource has increased [12–14]. One of the plants investigated over time for exhibiting significant biological properties is* Cinnamomum zeylanicum *Blume, commonly known as cinnamon. Several parts of the plant, such as the bark, leaves, flowers, fruits, and roots, have medicinal and culinary applications. The chemical composition of the materials obtained from the different parts of a plant exhibit considerable variation, resulting in different pharmacological effects [15–18].

Regarding the biological activity of* C. zeylanicum *Blume, its analgesic, antiseptic, anticancer, antispasmodic, coagulant, neuroprotective, hepatoprotective, gastroprotective, cardioprotective, and antimicrobial potentials, as well as its action for control of the scenic and lipid levels and reduction of the blood cholesterol concentration, have been described [15, 16, 18, 19]. Several studies presented further evidence of this plant's antimicrobial potential against fungi and gram-positive and gram-negative bacteria [20–24]. Despite demonstrations of the antifungal activity of* C. zeylanicum*, most studies have analyzed products extracted from the bark and used microorganisms in their planktonic phase.

Therefore, the aims of the present study were to describe the phytochemical profile of* C. zeylanicum *Blume and to investigate the* in vitro* antifungal activity, the mode of action, and the effect on the growth kinetics of* Candida* spp. in planktonic form and in biofilms of the essential oil (EO) extracted from leaves. In addition, the cytotoxic effect of this natural product on human peripheral blood cells and its phytochemical profile are described.

## 2. Materials and Methods

### 2.1. Test Product

Essential oil from the leaves of C*. zeylanicum *Blume (Ferquima Ind. and Com.,Vargem Grande, São Paulo, Brazil) is extracted using the hydrodistillation method and exhibiting a characteristic appearance, color and odor, specific gravity 1.043, and refractive index 1.533.

### 2.2. Microorganisms

The present study used* Candida *spp. reference strains* C. albicans *ATCC 60193,* C. albicans *ATCC 90029,* C. krusei *ATCC 34135, and* C. tropicalis *ATCC 750 from the American Type Culture Collection (ATCC) and* C. albicans *CBS 562 and* C. tropicalis *CBS 94 from the Centraalbureau voor Schimmelcultures (CBS). In addition, the following clinical strains were used, which were collected from the oral cavity, isolated, identified, and provided by the Laboratory of Clinical Mycology, Center of Health Sciences, Federal University of Paraíba:* C. albicans *LM01,* C. albicans *LM03,* C. albicans* LM04,* C. tropicalis* LM05,* C. tropicalis* LM07, and* C. tropicalis* LM08.

### 2.3. Phytochemical Analysis by Gas Chromatography-Mass Spectrometry (GCMS-QP2010 Ultra)

The volatile components profile was established using a Shimadzu GCMS-QP2010 gas chromatography-mass spectrometry device with Rtx-5MS capillary column, a stationary phase with 5% phenyl 95% dimethylpolysiloxane, 30 m of length, 0.25 mm of internal diameter, and 0.25 *μ*m of film thickness.

The initial temperature was set to increase from 60°C to 240°C (3°C/min). The run time was set to 60 minutes, and the temperature of the injector furnace was set to 250°C. Helium was used as the carrier gas (mobile phase) with a flow rate of 1.0 mL/min, a split ratio of 1:20, and an injection volume of 1 *μ*L. Component ionization was performed by means of electron impact at 70 eV with a 1.25 kV detector voltage. The spectrometer was used in the SCAN mode with a 40–500 a.m.u. mass range. The ion source temperature was 250°C, and the compounds were identified by comparing their mass spectra to the ones contained in the device database.

The C*. zeylanicum *Blume EO sample was injected in a concentration of 2 ppm, and hexane was used as the solvent. Chromatogram and mass spectra analysis were performed using the device library with integration parameters of width 3 and slope 2000.

### 2.4. Minimum Inhibitory Concentration (MIC)

The MIC was estimated by means of the microdilution technique on 96-well plates. For this purpose, 100 *μ*L of Sabouraud Dextrose Broth (SDB) medium (KASVI®, Kasv Imp e Dist de Prod p/Laboratorios LTDA, Curitiba, Brazil) was added to each well. C.* zeylanicum *Blume EO was added (100 *μ*L) to the first wells on the plates, following a serial dilution from 2,000 to 15.62 *μ*g/mL. The fungal strain inoculums were prepared and adjusted under the spectrophotometer to a concentration of 5.0 x 10^6^ colony forming units (CFU)/mL (absorbance 0.08 to 0.10 at 530 nm) and then diluted until reaching a final concentration of 2.5 x 10^3^ CFU/mL after having added 100 *μ*L to each well. Nystatin (Sigma-Aldrich, São Paulo–SP, Brazil) was used as a positive control in concentrations from 12 to 0.09 *μ*g/mL. Strain viability was assessed, as well as the sterility of the culture medium and of the Tween 80 (diluent of EO emulsion). The plates were incubated in an oven at 37°C for 24 h and then subjected to a visual reading [25]. To confirm the presence of viable microorganisms, 50 *μ*L of 2,3,5-triphenyltetrazolium chloride (TTC) was added to each well, and the plates were incubated for 24 h [26]. The test was performed in triplicate and repeated at three different time points.

### 2.5. Minimal Fungicidal Concentration (MFC)

Based on the results of the MIC assay, 50 *μ*L of culture from the wells that achieved the MIC and the two concentrations above it (MICx2 and MICx4) were used to seed Petri dishes containing Sabouraud Dextrose Agar (SDA) medium (KASVI®, Kasv Imp e Dist de Prod p/Laboratorios LTDA, Curitiba, Brazil). The dishes were incubated at 37°C for 48 h and a visual reading of the fungal growth was performed. The MFC was defined as the lowest concentration able to inhibit visible subculture growth. This test was performed in triplicate. To establish whether the tested EO had fungicidal or fungistatic activity, the ratio of the MFC to the MIC was calculated; activity was considered to be fungistatic when the MFC/MIC ≥ 4 and fungicidal when the MFC/MIC < 4 [27].

### 2.6. Essential Oil Mechanism of Action

#### 2.6.1. Sorbitol Test

The microdilution technique described in Section 2.4 was performed with and without sorbitol. The fungal inoculums were prepared using SDB medium (KASVI®, Kasv Imp e Dist de Prod p/Laboratorios LTDA, Curitiba, Brazil) with added sorbitol (D-sorbitol anhydrous-INLAB, São Paulo, SP, Brazil) to a final concentration of 0.8 M. Sorbitol acts as an osmotic protector; therefore, a reduction in the action of the tested substance in its presence is indicative of action on the fungal cell wall. The plates were incubated at 37°C for 48 h before being read [28, 29]. Caspofungin (caspofungin diacetate, Sigma-Aldrich, São Paulo, SP, Brazil) was used as a positive control [30, 31]. This test was performed in triplicate. The mechanism of action was established as involving the cell wall when the MIC attained by the test substance in the presence of sorbitol was higher than the one attained in the absence of sorbitol.

#### 2.6.2. Ergosterol Test

To establish whether the mechanism of action of the tested substance was related to the ergosterol present in the cell membrane, the microdilution technique described in Section 2.4 was performed with and without exogenous ergosterol (Sigma-Aldrich, São Paulo, SP). The* C. albicans* ATCC 60193 and* C. tropicalis* ATCC 750 inoculums were prepared in SDB medium with added ergosterol (at 400 *μ*g/mL). The plates were incubated at 37°C for 48 h before being read [28, 29]. Nystatin (Sigma-Aldrich, São Paulo, SP) and amphotericin B (Sigma-Aldrich, São Paulo, SP) were used as positive controls [30, 31]; the involvement of the cellular membrane was established as the mechanism of action when the MIC attained by the test substance in the presence of ergosterol was higher than the one attained in the absence.

### 2.7. Assessment of the Inhibition Kinetics of* C. zeylanicum *Blume EO on Fungal Growth

The interference of the test substance with the growth and multiplication of* C. albicans* ATCC 60193 cells was studied by means of the CFU counting method [32, 33]. The effect of the test substance concentrations corresponding to MIC, MICx2, and MICx4 on fungal growth was assessed at various time points: T0 (baseline), T1 (1 hour), T2 (2 hours), T6 (6 hours), T8 (8 hours), T12 (12 hours), and T24 (24 hours).

The same protocol of microdilution on 96-well plates was used to grow the fungus under the effect of the test substance [25]. Next, Petri dishes containing SDA medium (KASVI®, Kasv Imp e Dist de Prod p/Laboratorios LTDA, Curitiba, Brazil) were seeded with 10 *μ*L of the well contents at the present time points. The dishes were incubated at 37°C for 24 h before the CFU counting, which was determined visually, and the values were transformed into log_10_ CFU/mL and presented as a fungal cell death curve. Nystatin was used as positive control; the growth of the tested strains and sterility of the culture medium were assessed.

### 2.8. Assessment of the Antimicrobial Activity of* C. zeylanicum *Blume EO on* Candida* spp. Monospecies and Multispecies Biofilm

This assay investigated the effect of* C. zeylanicum *Blume EO on two stages of biofilm development: during formation and at maturity (48 h). Three types of biofilm were used in these cases:* C. albicans* ATCC 60193 monospecies,* C. tropicalis *ATCC 750 monospecies, and* C. albicans *ATCC 60193 and* C. tropicalis *ATCC 750 multispecies.

#### 2.8.1. Assessment of the Antimicrobial Activity of* C*.* zeylanicum* Blume EO on* Candida* spp. Biofilm Formation

The effect of the test EO on biofilm formation was assessed on 96-well flat-bottom plates (CRAL Artigos para Laboratório LTDA, Cotia-SP, Brazil). Each well received 100 *μ*L of SDB medium (KASVI®, Kasv Imp e Dist de Prod p/Laboratorios LTDA, Curitiba, Brazil).* C. zeylanicum *Blume EO solution was added (100 *μ*L) to the first wells on the plates and subjected to serial dilution from 1,000 to 7.81 *μ*g/mL. Nystatin was used as the positive control in concentrations from 100 to 0.78 *μ*g/mL. Next, the inoculums, which were prepared in the SDB medium with 2% sucrose and adjusted under the spectrophotometer (BioPet Technologies–722G) to a concentration of 2.5 x 10^5^ CFU/mL, were transferred to the plates. A volume of 100 *μ*L of inoculum was used for the monospecies biofilm, and a volume of 50 *μ*L of each strain (*C. albicans* ATCC 60193 and* C. tropicalis *ATCC 750) was used for the multispecies biofilm. The plates were incubated at 37°C for 48 h.

#### 2.8.2. Biofilm Biomass Quantification

Once the incubation period was complete, the culture medium was taken from the plates, and the nonadhered cells were removed by rinsing the wells twice with 200 *μ*L of phosphate buffered saline (PBS), followed by drying at room temperature for 45 minutes. A total of 100 *μ*L of 0.4% crystal violet solution was added to the wells and left in contact with the biofilm for 45 minutes. The wells were then rinsed three times with 200 *μ*L of distilled water and immediately destained with 200 *μ*L of 95% ethanol for 45 minutes. Finally, 100 *μ*L of the destained solution was transferred to another plate, and the absorbance was read using a GloMax®-Multi Detection System–Promega plate reader at 600 nm [34].

The EO absorbance and growth control values were used to calculate the percent of biofilm formation inhibition; growth control was considered 100% of the fungal formation. The test was performed in triplicate. The samples used for the sterility assessment were not added to the cell suspension, and the ones used for growth control, as well as the fungal strains corresponding to each biofilm type, were added to the culture medium.

#### 2.8.3. Assessment of the Antimicrobial Activity of* C. zeylanicum* Blume EO on Reduction of* C. albicans* Mature Biofilm

A total of 200 *μ*L of the fungal suspension corresponding to each type of the assessed biofilm grown in the SDB medium (KASVI®, Kasv Imp e Dist de Prod p/Laboratorios LTDA, Curitiba, Brazil), which was supplemented with 2% of sucrose and adjusted to a concentration 2.5 x 10^5^ CFU/mL, was added to the 96-well flat-bottom plates. The plates were incubated at 37°C for 48 h to allow for mature biofilm formation. Next, the culture medium was removed from the plates, and the wells were rinsed with 200 *μ*L of PBS so that only adhered biofilm remained in the plates. The* C. zeylanicum *Blume EO solution (100 *μ*L) was added in concentrations ranging from 1,000 to 7.81 *μ*g/mL to the plates containing the mature biofilm. Nystatin was used as a positive control in concentrations from 100 to 0.78 *μ*g/mL. The samples were divided into two groups according to the duration of the incubation period and also to the duration of their exposure to the EO: G1 (24-h incubation in an oven at 37°C) and G2 (48-h incubation in an oven at 37°C). Once the time allotted for incubation was over, the mature biofilm reduction was quantified using the technique described in Section 2.8.2.

### 2.9. Assessment of* C. zeylanicum* Blume EO Cytotoxicity on Human Peripheral Blood Cells

#### 2.9.1. Ethical Issues and Volunteers

The study was approved by the research ethics committee of Federal University of Paraíba, ruling no. 1869951. Blood for isolation of the peripheral blood mononuclear cells (PBMCs) was donated by five volunteers, who gave written consent by signing an informed consent form. The inclusion criteria were healthy individuals, who had no history of infectious or metabolic diseases and were not using immunomodulating drugs.

#### 2.9.2. Hemolysis Test

The hemolytic activity of* C. zeylanicum* Blume EO was investigated using the method of [35]. EO solution was prepared using four solvents: water, water with neutralized pH, serum, and serum with neutralized pH. One hour after the EO was added to the wells containing the red blood cells, 70 *μ*L of the supernatant was transferred to a 96-well flat-bottom plate, and the absorbance was read using a GloMax®-Multi device at 560 nm. Red blood cells with added distilled water and normal saline were used as positive and negative controls, respectively. The percent hemolysis was calculated using the following equation:

(1) $$\begin{array}{c} \%\;\;hemolysis=\frac{AA\text{ - }AN}{AP\text{ - }AN}\times 100, \end{array}$$

where AA, AP, and AN correspond to the sample absorbance, positive control absorbance, and negative control absorbance, respectively. According to the index recommended by [36], percentages under 5% should be considered as acceptable hemolysis.

#### 2.9.3. PBMC Cell Viability against the* C. zeylanicum* Blume EO Assay

Heparinized total blood was collected through venipuncture and centrifuged for 8 h with Ficoll-Paque ™ specific gravity gradient 1077 (GE Healthcare). The PBMCs were collected, rinsed three times with phosphate buffer saline, and counted via trypan blue exclusion (Sigma-Aldrich, St. Louis, USA) in a Neubauer chamber. The cells were resuspended in aliquots of 2 x 10^6^ PBMCs/mL in RPMI 1640 medium (GIBCO, Life Technologies, UK) containing 10% of inactivated fetal bovine serum (GIBCO, USA),followed by the addition of phytohemagglutinin in a concentration of 5 *μ*g/mL (PHA-P, Sigma-Aldrich, St. Louis, MO, USA) [37].

Black, 96-well, polystyrene plates (Greiner Bio-One, USA) were used to incubate 100 *μ*L of PBMC suspension and* C. zeylanicum* Blume EO (31.25–2,000 *μ*g/mL) at a ratio of 1:1 in culture medium at 37°C, in a humidified atmosphere, and with 5% CO_2_ for 24 h. Next, the cell viability was measured using the alamarBlue® fluorescence protocol (Bio-Rad, Hercules, CA, USA). The percent viability was calculated as follows:

(2) %  cytotoxicity=FI  590  of  treated  samplesFI  590  of  non-treated  cells100,

where FI 590 = the fluorescence intensity at 590-nm emission (560-nm excitation).

### 2.10. Statistical Analysis

Exploratory analysis was performed first to establish the most adequate statistical test. Significant differences between the controls and test substance in the kinetic, biofilm, and PBMC cytotoxicity assays were assessed with the Kruskal-Wallis test followed by Dunn's post hoc test (*α* = 0.05) using GraphPad Prism version 7.0 (San Diego, CA, USA).

## 3. Results

### 3.1. Chromatographic Profile and Identification of Compounds in* C. zeylanicum *Blume EO

The chemical composition of* C. zeylanicum *Blume EO is described in Table 1. Twenty-six components were identified, corresponding to 100% of the EO composition. Eugenol was the largest component (68.96%), whereas the concentration of all others was under 3.5%. Analysis demonstrated the presence of volatile compounds, particularly aromatic and terpenic compounds.

### 3.2. Determination of the MIC and MFC

The MIC and MFC values corresponding to the* C. zeylanicum *Blume EO and the standard antifungal nystatin are described in Table 2. Relative to the EO, the MIC and MFC ranged from 125 to 1,000 *μ*g/mL, with* C. krusei *ATCC 60193 being the most resistant strain. The vehicles used for the EO emulsion (distilled water and Tween 80) did not affect the growth of the fungal strains. The MFC/MIC ratio showed that the EO had fungicidal effect on all the* Candida* species tested [27].

### 3.3. *Cinnamomum zeylanicum *Blume EO Mechanism of Action

The values described in Table 3 show that the antifungal properties of the EO are related to the fungal cell wall biosynthesis pathways, as the MIC in the presence of sorbitol was higher compared to the MIC in the absence in all the tested strains, except for* C. albicans *LM04. For caspofungin, which was used as a positive control, the MIC was at least four times higher in the presence of sorbitol in all the tested strains.

The results of the test that assessed the MIC in the presence of the ergosterol suggest that, in the case of species* C. tropicalis*, EO has weak action on the ion permeability of the cell membrane. This effect was not detected in the* C. albicans* strain, as the presence of ergosterol was not associated with an increase in the MIC. The nystatin and amphotericin B controls confirmed the previously established mechanism of action (Table 4).

### 3.4. Assessment of the Inhibition Kinetics of* C. zeylanicum *Blume EO on Fungal Growth

**Figure 1:**
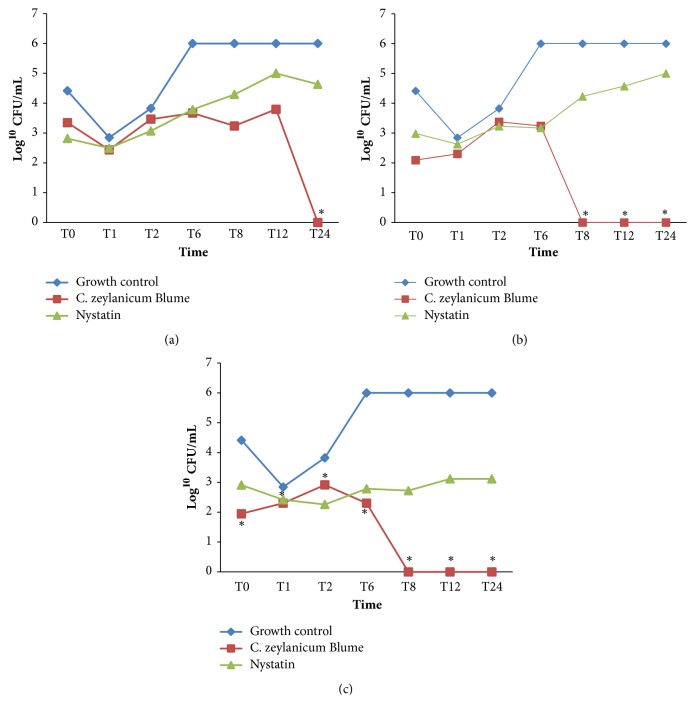
*Cinnamomum zeylanicum *Blume EO (250 *μ*g/mL) and nystatin interference with the growth and multiplication of* C. albicans* ATCC 60193. Concentrations: (a) MIC: cinnamon (250 *μ*g/mL)/nystatin (0.375 *μ*g/mL); (b) MIC x 2: cinnamon (500 *μ*g/mL)/nystatin (0.750 *μ*g/mL); (c) MIC x 4 cinnamon (1,000 *μ*g/mL)/nystatin (1.5 *μ*g/mL). *∗*Significant CFU reduction (Kruskal-Wallis test followed by Dunn's* post hoc* test, *P* < 0.05).

*Cinnamomum zeylanicum *Blume EO interfered with the growth of* C. albicans* ATCC 60193 in all the tested concentrations (250 *μ*g/mL, 500 *μ*g/mL, and 1,000 *μ*g/mL), inducing a significant reduction in fungal growth, as shown by the Kruskal-Wallis test followed by Dunn's post hoc test (*α* = 0.05). At the MIC level, the fungus was completely eliminated after 24-h exposure to EO; at the CIMx2 and CIMx4 levels, the same effect was achieved after 8 h (Figure 1).

### 3.5. *Cinnamomum zeylanicum* Blume EO's Effect on* Candida* spp. Monospecies and Multispecies Biofilm

**Figure 2:**
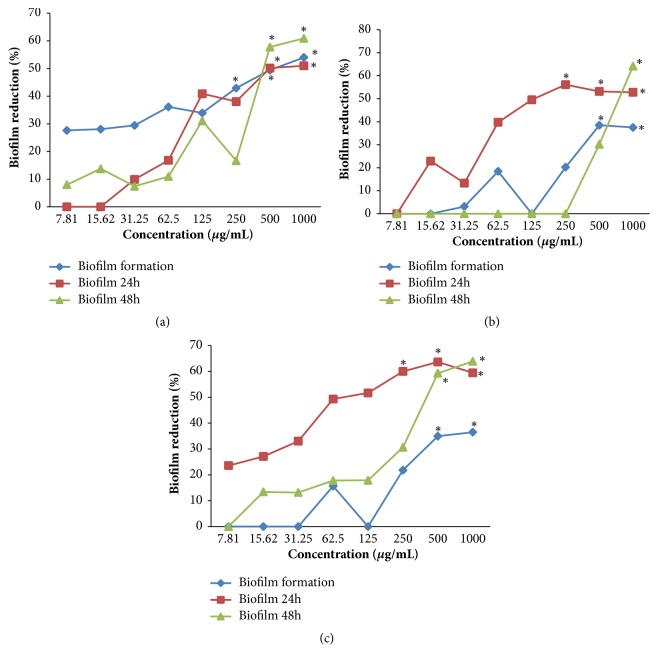
*Cinnamomum zeylanicum* Blume EO inhibitory effect on biofilm formation and mature biofilm reduction (G1: 24-h contact with EO; G2: 48-h contact with EO) corresponding to (a)* Candida albicans* ATCC 60193; (b)* Candida tropicalis *ATCC 750; and (c)* Candida *spp. multispecies (*Candida albicans *ATCC 60193 and* Candida tropicalis* ATCC 750). *∗*Significant biofilm reduction (Kruskal-Wallis test followed by Dunn's post hoc test, p < 0.05).

**Figure 3:**
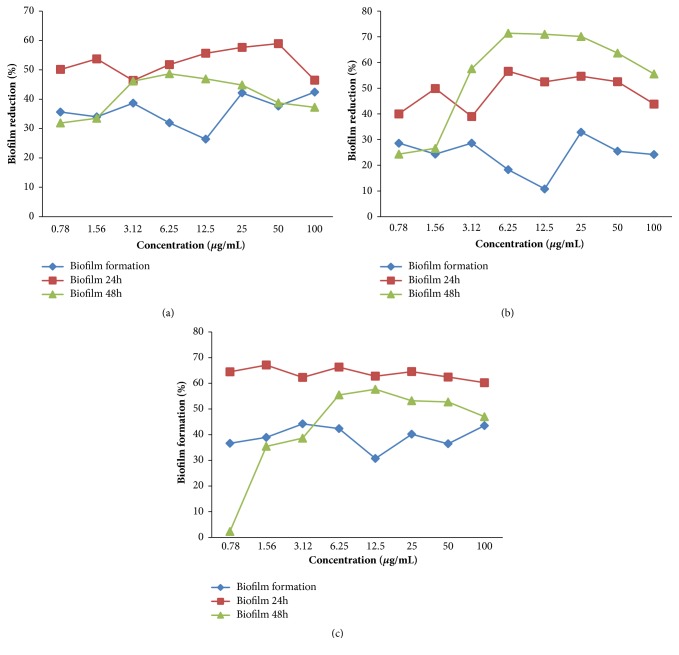
Nystatin inhibitory effect on biofilm formation and mature biofilm reduction (G1: 24-h contact with EO and G2: 48-h contact with EO) corresponding to (a)* Candida albicans* ATCC 60193; (b)* Candida tropicalis *ATCC 750; and (c)* Candida *spp. multispecies (*Candida albicans *ATCC 60193 and* Candida tropicalis* ATCC 750). *∗*Significant biofilm reduction (Kruskal-Wallis test followed by Dunn's post hoc test, p < 0.05).

*Cinnamomum zeylanicum* Blume OE inhibited the formation of the* Candida* spp. monospecies and multispecies biofilm and reduced the mature biofilm in both tested groups (24 h and 48 h). The best results concerning mature biofilm reduction were detected after 24 h of exposure, as the lowest concentration of EO (250 *μ*g/mL) was able to significantly reduce* C. tropicalis *ATCC 750 monospecies biofilm (p = 0.0017) and* Candida *spp. multispecies biofilm (p = 0.0081). A concentration of 500 *μ*g/mL induced a significant reduction in all phases of biofilm development in all biofilm types, except for the* Candida tropicalis *ATCC 750 mature biofilm after 48 h of exposure (Figure 2). The results corresponding to nystatin are shown in Figure 3.

### 3.6. Cytotoxicity

**Figure 4:**
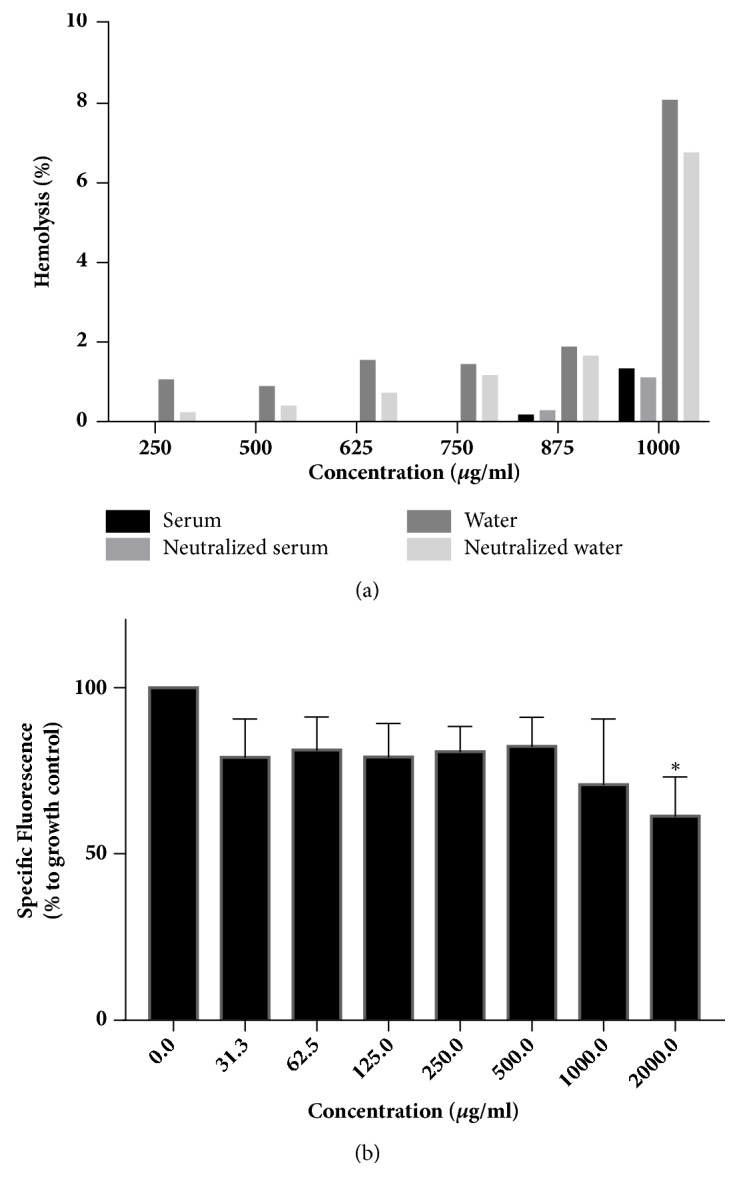
Cytotoxicity of* C. zeylanicum* Blume EO. (a) Hemolytic activity of the different concentrations of* C. zeylanicum* Blume EO on human red blood cells. (b) Percent PBMC viability after treatment with different concentrations of* C. zeylanicum* Blume EO. *∗*Significant reduction in cell viability (Kruskal-Wallis test followed by Dunn's post hoc test,* p *< 0.05).

The percent hemolysis induced by EO is depicted in Figure 4. Up to a concentration of 750 *μ*g/mL, the EO diluted in the serum and neutralized serum exhibited a protective effect on human red blood cells. In concentrations of 875 *μ*g/mL and 1,000 *μ*g/mL, the highest percentages of hemolysis were 2% and 8%, respectively, which correspond to the EO solution in nonneutralized water.

The* C. zeylanicum* Blume EO did not induce a significant reduction of the human PBMC viability in concentrations of up to 1,000 *μ*g/mL; neither was dose-dependency detected up to this concentration. A significant reduction compared to the control was detected with concentrations of 2,000 *μ*g/mL only (Figure 4).

## 4. Discussion

The increasing limitations of the antifungals currently used for treatment of candidiasis in association with the high prevalence of disease and the demonstrated antimicrobial potential of plant essential oils [38–40] led to the present study. In this study, the antifungal effect of* C. zeylanicum *Blume EO on* Candida *spp. planktonic cells and biofilm and the possible mechanisms of action involved in this effect were investigated.

The results of the chemical analysis of the EO extracted from* C. zeylanicum *Blume leaves in the present study agree with the findings by previous authors, which indicate that eugenol is its largest component (68.96%) [38, 41], whereas the other 25 chemical compounds appear in smaller concentrations.

Natural products are considered powerful inhibitors of microbial activity when the MIC is equal to or lower than 500 *μ*g/mL [42]. Consistently, the results of the present study show that* C. zeylanicum* Blume EO had powerful antifungal activity on all the tested strains. The only exception was* C. krusei *ATCC 3413, for which the effect might be rated as moderate, which is also the case with the available antifungals [43]. The MFC/MIC ratio suggests that the tested EO had a fungicidal effect [27] on both the reference (ATCC and CBS) and clinical strains. Reports in the literature corroborate the effect of* C. zeylanicum* EO on* Candida* spp. [39, 41, 44–46]. However, these studies exhibit significant variability as to the* Cinnamomum* species and plant material used and the methods used to investigate fungal sensitivity, whereas some of them do not indicate from which plant part the EO was extracted. These differences make comparison difficult, as one plant might produce oils with different main components as a function of the plant part used, and different species of the same genus produce EOs with variable chemical composition, resulting in different biological effects [15, 16]. In addition, clinical* Candida* strains that are multiresistant to the drugs that are widely employed in clinical practice demonstrated susceptibility to* C. zeylanicum *in other studies [47, 48].

The antifungal activity detected at the MIC and MFC levels might be attributed to the complex combination of volatile components, particularly to the EOs, which account for the different biological activities in humans, animals, and plants. Such activity might correspond to two or three main components, i.e., the ones with higher concentration compared to other components [49, 50].

Notably, previous studies corroborate the antifungal activity of eugenol, the largest component of the EO used in the present study, on* Candida* spp. planktonic cells, biofilm under formation, and mature biofilm [51–53]. One study that assessed the correlation between the antifungal activity and chemical components of* C. zeylanicum* Blume EO attributed the strong antifungal effect of this EO on* C. albicans,* C.* parapsilosis,* C.* tropicalis, *and C.* glabrata *strains (MIC from 0.31 to 0.63 *μ*g/mL) to the eugenol [41]. However, when the same authors compared the MIC of EO and eugenol individually, they found that the MIC was slightly higher for the latter, which suggests that other EO components, even if in small concentrations, act synergically, thus contributing to the antifungal effect of the EO. Therefore, eugenol can be inferred to be the main component responsible for the antifungal effect detected in the present study, but that other components, such as cinnamaldehyde, linalool, benzyl benzoate, alpha-pinene, and beta-pinene, must have also contributed to that effect, as the antifungal activity of each individual compound has been demonstrated [41, 46, 54, 55].

To understand better the antifungal effect detected with the MIC and MFC tests, the effect of the EO on the fungal growth kinetics was assessed. This test showed that for the three concentrations assessed (MIC, MICx2, and MICx4), the effect of the EO on* C. albicans* began to be perceptible after 8-h exposure and that a 24-h exposure was sufficient for the antifungal effect of the EO to occur. No studies assessing the effect of EO extracted from* C. zeylanicum* Blume leaves on* Candida* growth and development could be found in the literature.

The study of the mechanism of action of antifungals is a relevant strategy for limiting the emergence of resistance to the drugs currently available, as well as for developing more powerful and safe drugs against infection [56, 57]. However, no studies assessing the mechanism of action of* C. zeylanicum *Blume EO against* Candida* spp. could be found in the literature. The results of the present study indicate that the EO antifungal action seems to be related to the fungal cell wall biosynthesis pathways. However, the tested substance cannot be concluded to interfere with the cell membrane ion permeability through binding to ergosterol because the MIC of only one of the assessed species (*C. tropicalis *ATCC 750) increased twice in the presence of exogenous ergosterol. Considering the discrete increase of MIC detected in the presence of an osmotic protector (sorbitol) and the complex composition of EO, the antifungal activity of EO might involve other mechanisms of action, in addition to the ones addressed in the present study. Because the antifungal effect of eugenol, the main component of the tested EO, on* Candida* spp. is related to ergosterol biosynthesis, a study of this pathway might provide promising hypotheses for future studies [58].

In addition to the ability of the* C. zeylanicum* Blume EO to act on* Candida* spp. planktonic cells, an interesting antifungal potential was detected relative to the* Candida* spp. biofilm in the various stages of formation. At concentrations of 500 *μ*g/mL, the EO reduced the* Candida* spp. biofilm formation by 34.94% to 49.42%. For the mature biofilm, the EO at a concentration of 500 *μ*g/mL reduced all the tested biofilm types by at least 50%, except for the* C. tropicalis* biofilm, which exhibited a decrease of 30.2% after 48-h exposure to EO. These findings suggest that the potential of* C. zeylanicum *Blume EO to reduce mature biofilm is superior to its ability to inhibit the formation of the microbial community as biofilm. On these grounds, the tested EO might be used as an antifungal for patients affected with candidiasis, i.e., already exhibiting formed biofilm, rather than as a preventive agent, in which case it would play a role in the inhibition of the microorganisms' adherence.

Notably, at a concentration of 250 *μ*g/mL and after 24 h of exposure, the EO induced a statistically significant (p < 0.05) reduction of the mature biofilm in two out of the three types of investigated biofilms (*C. tropicalis* monospecies and* Candida* spp. multispecies). Similarly, all the stages of* Candida* biofilm formation were sensitive to EO at concentrations of 500 *μ*g/mL, suggesting the use of this concentration in the development of medicines based on* C. zeylanicum* Blume EO.

Few studies exist in the literature on the antimicrobial effect of* C. zeylanicum* Blume EO. A single study assessed its action on* Candida,* and no study using* C. zeylanicum* Blume EO extracted from the plant leaves could be located [24, 38, 59, 60]. [59] analyzed the effect of* C. zeylanicum* Blume EO on* C. parapsilosis *and* C. albicans *biofilm formation and reduction and found the full inhibition of biofilm formation at concentrations of 250 *μ*g/mL and 125 *μ*g/mL, respectively. Mature biofilm reduction (after 24-h formation) was analyzed based on the minimum biofilm reduction concentration values, which were 1,000 *μ*g/mL and 2,000 *μ*g/mL for* C. parapsilosis *and* C. albicans, *respectively. Although the authors did not mention the plant part used for the EO production, the extraction was presumably made from the bark because phytochemical analysis showed that cinnamaldehyde was the main component [15, 16]. This component might account for the differences found relative to the present study, in which the EO was extracted from the plant leaves, and eugenol was the largest component. Eugenol was assessed for its ability to inhibit* C. albicans* biofilm formation and reduce the mature biofilm (after 24-h formation); the results showed that it could inhibit biofilm development by 70% when used at a concentration of 500 *μ*g/mL and that it could reduce the mature biofilm at concentrations of 1,000 *μ*g/mL [52].

Susceptibility of the biofilms of other microorganisms, such as* Enterococcus faecalis* [24],* Escherichia coli*,* Staphylococcus aureus *[38], and* Pseudomonas aeruginosa *[60], is also reported in the literature. Although very few, the studies that assessed the effect of* C. zeylanicum *EO on microbial biofilm had promising outcomes vis-à-vis its possible use in infectious diseases.

In the part of the study on the EO cytotoxicity on human red blood cells, the percent hemolysis found in the samples diluted in the neutralized and nonneutralized serum was dismissible in all the tested concentrations according to the reference values recommended by [36], according to whom values under 5% are dismissible. Based on the same criterion, only the EO diluted in the distilled water at a concentration of 1,000 *μ*g/mL induced more than 5% hemolysis. In all the tested concentrations, the neutralized solvent induced less hemolysis, which points to the relevance of the neutralization of solutions, as the occurrence of hemolysis in the living body is undesirable. No study assessing the hemolytic effect of* C. zeylanicum *Blume EO could be found in the literature for comparison with the results of the present study.

The viability of human peripheral blood cells had no dose-dependency relation with the EO effect up to a concentration of 1,000 *μ*g/mL, with the cell viability reduction being approximately 30%. The EO at concentrations of 2,000 *μ*g/mL significantly reduced cell viability; however, the results of the present study indicate that the antifungal effect of the EO occurred in concentrations at least four times lower than the one that induced a toxic effect on the PBMCs. In addition, other studies that assessed the cytotoxic effect of* C. zeylanicum *found compatibility among the various cell lines, with the percent cell viability being at least 80% [24]. However, those studies used the plant bark, whereas the present study used leaves as the source of the EO extraction, which once again emphasizes the originality of the latter.

Although countless* in vitro* studies have used natural products for the treatment of candidiasis, with optimistic results, currently, the level of scientific evidence is low due to significant methodological differences in the design of the studies, the choice of extracts, and the concentrations used in the tests [61]. That is, much research has been conducted, but little advances have been achieved regarding the treatment of the disease. The* C. zeylanicum* Blume EO used in the present study is a natural product with potential for clinical application in the treatment of candidiasis. More thorough studies, however, including* in vitro* investigations of toxicity and clinical trials, are needed to develop an antifungal agent based on* C. zeylanicum* Blume EO.

## 5. Conclusion

The EO extracted from* C. zeylanicum* Blume leaves has eugenol as its largest component, has antifungal action on* Candida* spp. planktonic cells and monospecies and multispecies biofilm, acts on the cell wall biosynthesis, and is poorly cytotoxic against human red blood cells and PBMCs. The evidenced properties of the EO extracted from* C. zeylanicum* Blume leaves encourage the performance of phase I and II clinical studies to validate its use in oral fungal infections.

## Figures and Tables

**Table 1:** Identification of *C. zeylanicum *Blume EO components by means of GC-MS.

**Retention time (min)**	**Compound name**	**Area (**%**)**
**5.942**	Alpha-pinene	1.12
**6.178**	Hydroperoxide, 1-ethylbutyl	0.50
**6.370**	Camphene	0.24
**6.450**	Hydroperoxide	0.62
**7.189**	Beta-pinene	0.32
**8.037**	Alpha-phellandrene	0.94
**8.725**	p-*Cymene*	1.00
**8.890**	Beta-phellandrene	0.65
**11.566**	Linalool	2.70
**19.084**	(*E)-Cinnamaldehyde*	1.17
**19.898**	5-(2-propenyl)-1,3-Benzodioxole	1.30
**23.029**	Eugenol	68.96
**23.758**	*Alpha-copaene*	0.60
**25.667**	Caryophyllene	3.54
**26.714**	2-Propen-1-ol, 3-phenyl-, Acetate	1.30
**27.127**	*Alpha-humulene*	0.61
**30.176**	*Eugenyl acetate*	2.04
**32.516**	*Caryophyllene oxide*	1.01
**35.798**	1-Dodecanol	0.61
**39.431**	Benzyl benzoate	3.29
**40.895**	Methoxyacetic acid, dodecyl ester	0.46
**43.306**	n-Heptadecanol-1	0.97
**46.185**	n*-Hexadecanoic acid*	2.18
**50.043**	1-Octadecanol	1.74
**52.552**	Eicosanoic acid	1.33
**58.368**	9-Octadecenamide, (Z)-	0.81
		100.00

**Table 2:** Antifungal activity of *C.zeylanicum *Blume EO on *Candida *spp. (MIC and MFC values expressed as *μ*g/mL).

**Strain**	***C. zeylanicum *Blume EO**			**Nystatin**		
	**MIC**	**MFC**	**MIC/MFC ratio**	**MIC**	**MFC**	**MIC/MFC ratio**
***C. albicans *CBS 562**	250	250	1	0.75	1.5	2
***C. albicans *ATCC 60193**	250	250	1	0.375	0.75	2
***C. krusei *ATCC 3413**	1000	1000	1	3	3	1
***C. tropicalis* CBS 94**	250	500	2	1.5	1.5	1
***C. tropicalis* ATCC 750**	250	500	2	0.75	0.75	1
***C. albicans *ATCC 90029**	125	125	1	*∗*	*∗*	*∗*
***C. albicans *LM01**	250	250	1	*∗*	*∗*	*∗*
***C. albicans *LM03**	250	250	1	3	6	2
***C. albicans* LM04**	500	500	1	3	3	1
***C. tropicalis* LM05**	250	250	1	*∗*	*∗*	*∗*
***C. tropicalis* LM07**	250	250	1	3	6	2
***C. tropicalis* LM08**	62.5	125	2	<0.093	*∗*	*∗*

*∗* Not tested

**Table 3:** MIC values of *C. zeylanicum *Blume EO and caspofungin in the presence and absence of sorbitol (0.8 M).

**Strain**	***C. zeylanicum *EO**		**Caspofungin**	
	**MIC (** ***μ*** **g/mL) **	**MIC with sorbitol (** ***μ*** **g/mL)**	**MIC (** ***μ*** **g/mL)**	**MIC with sorbitol (** ***μ*** **g/mL)**
***C. albicans* CBS 562**	250	500	< 0.039	>5
***C. albicans* ATCC 60193**	250	500	< 0.039	> 5
***C. albicans* LM03**	250	500	<0.078	5
***C. albicans* LM04**	500	500	<0.078	10
***C. tropicalis *LM07**	250	500	<0.078	10
***C. tropicalis *LM08**	62.5	500	<0.078	1.25

**Table 4:** Effect of exogenous ergosterol (400 *μ*g/ml) on the MIC of *C. zeylanicum *Blume EO against strains *C. albicans *CBS 562 and* C. tropicalis *ATCC 750 (values expressed as *μ*g/mL).

**Strain**	***C. zeylanicum *EO**		**Nystatin**		**Amphotericin B**	
	**MIC**	**MIC with ergosterol**	**MIC**	**MIC with ergosterol**	**MIC**	**MIC with ergosterol**
***C. albicans *ATCC 60193**	250	250	0.375	3	<0.5	31.25
***C. tropicalis *ATCC 750**	250	500	0.75	6	<0.5	15.62

## Data Availability

All the datasets used/or analyzed during the current study are available from the corresponding author on reasonable request.
